# Toward system-level understanding of baculovirus–host cell interactions: from molecular fundamental studies to large-scale proteomics approaches

**DOI:** 10.3389/fmicb.2012.00391

**Published:** 2012-11-09

**Authors:** Francisca Monteiro, Nuno Carinhas, Manuel J. T. Carrondo, Vicente Bernal, Paula M. Alves

**Affiliations:** ^1^Animal Cell Technology Unit, Instituto de Biologia Experimental e TecnológicaOeiras, Portugal; ^2^Animal Cell Technology Unit, Instituto de Tecnologia Quimica e BiológicaOeiras, Portugal; ^3^Departamento de Química, Faculdade de Ciências e Tecnologia, Universidade Nova de LisboaCaparica, Portugal; ^4^Departamento de Bioquímica y Biología Molecular B e Inmunología, Facultad de Química, Regional Campus of International Excellence “Campus Mare Nostrum”, Universidad de MurciaMurcia, Spain

**Keywords:** baculovirus, virus–host interactions, cytoskeleton, apoptosis, stress response, proteomics

## Abstract

Baculoviruses are insect viruses extensively exploited as eukaryotic protein expression vectors. Molecular biology studies have provided exciting discoveries on virus–host interactions, but the application of omic high-throughput techniques on the baculovirus–insect cell system has been hampered by the lack of host genome sequencing. While a broader, systems-level analysis of biological responses to infection is urgently needed, recent advances on proteomic studies have yielded new insights on the impact of infection on the host cell. These works are reviewed and critically assessed in the light of current biological knowledge of the molecular biology of baculoviruses and insect cells.

## BRIEF INTRODUCTION TO THE BACULOVIRUS–INSECT CELL SYSTEM

Baculoviruses are rod-shaped viruses with double-stranded DNA genomes. They infect arthropods, mainly insects, a feature that encouraged their usage as ecologically friendly biopesticides (Miller, 1997). Later on, baculoviruses started to be exploited as viral vectors for eukaryotic protein expression, which quickened the pace of their characterization at the cellular and molecular levels (Possee, 1993). The best-studied member of this family, *Autographa californica* multicapsid nucleopolyhedrovirus (*Ac*MNPV), encodes for 150 genes and has its own genome completely sequenced. The cell lines used for *Ac*MNPV propagation are derived from the pupal ovarian tissue of the fall armyworm *Spodoptera frugiperda*, being the *Sf*9 clonal isolate the most frequently used. Insect cells possess several advantages as protein expression factories. They grow in suspension without serum supplementation, are able to perform post-translational modifications and the scale-up of cultures is quite straightforward (Ikonomou et al., 2003). However, the production potential of insect cells has been hampered by the so called “cell density effect,” i.e., the drop in specific productivity when cells are infected with baculovirus at high cell concentration. As *Sf*9 cells attain high densities, central metabolism suffers a general down-regulation (Bernal et al., 2009). Viral infection induces a multi-level response in the host, during which a vast number of intracellular pathways are activated/deactivated (i.e., regulated) and profound metabolic changes occur. These changes are ultimately responsible for the production performance of the system. A deeper understanding of such phenomena will certainly allow for the rational design of strategies for bioprocess optimization. The genome of *S. frugiperda *remains unsequenced, which limits the extent to which high-throughput (genome-scale) technologies can be applied. To date, only few proteomic-based high-throughput studies concerning the impact of baculovirus infection on their host cells have been pursued. The aim of this review is to summarize the state of the art in the molecular biology of the baculovirus replication and host–virus interaction in this system. The implications of these mechanisms for proper infection and their potential use for the improvement of baculoviruses-based applications such as protein production and use as gene therapy vectors will be highlighted.

## BACULOVIRUS LIFE CYCLE: FROM *IN VIVO* TO *IN VITRO* CULTURES

Baculoviruses have a biphasic replication cycle in the insect host, involving the formation of two types of virions which are produced in different phases of the infection process and have different roles: the occlusion-derived virions (ODVs), adapted for stability outside the insect host, and the budded virions (BVs), non-occluded and responsible for the systemic, cell-to-cell dissemination of the virus within the insect. Specific roles, morphology, and functionality of both virion types are described in more detail below. Moreover, the virus life cycle is temporally divided into three consecutive phases (immediate-early/early; late and very late) regarding gene expression programming (Passarelli and Guarino, 2007). Specific constraints and events of each phase are summarized along the text.

Baculovirus infection starts when insect larvae ingest the occlusion bodies (Keddie et al., 1989). These are forms resistant to environmental factors. ODVs are embedded in a proteinaceous matrix mostly composed of the very late expressed protein polyhedrin. When facing the alkaline conditions in the insect midgut, the occlusion body dissolves and releases the ODVs, and the polyhedrin matrix is in turn degraded by proteinases present in the gut or associated with the virions (Wang and Granados, 1997). The replicative cycle begins when ODVs infect the midgut columnar epithelial cells. ODVs possess a set of specific envelope-associated proteins, called *per os* infectivity factors (*pif*), which mediate virion-specific binding to receptors located at the membrane of midgut epithelial cells (Horton and Burand, 1993; Kikhno et al., 2002). To date, six proteins have been identified as members of this family, P74 (PIF-0), PIF-1, PIF-2, PIF-3, PIF-4, and PIF-5 (ODV-E56; Faulkner et al., 1997; Kikhno et al., 2002; Pijlman et al., 2003; Ohkawa et al., 2005; Fang et al., 2009a; Harrison et al., 2010; Sparks et al., 2011). P74, PIF-1, and PIF-2 were shown to co-localize at the ODV envelope (Faulkner et al., 1997; Kikhno et al., 2002; Fang et al., 2006, 2009a; Harrison et al., 2010) and, together with PIF-3, constitute the core PIF complex that mediates viral entry. Afterward, viral entry occurs via a non-endocytic pathway through membrane fusion of the virion envelope with microvilli of epithelial cells, accompanied by the release of nucleocapsids into the cytoplasm. Nucleocapsids then migrate to the nucleus in a process that involves actin polymerization (Ohkawa et al., 2010).

Once having reached the nucleus, the host cell RNA polymerase-dependent transcription of viral immediate-early/early genes initiates (0–6 h post-infection, hpi). This set of genes encodes mainly for transactivators essential for both subsequent viral gene expression and subversion of host cell activity (Passarelli and Miller, 1993). The transition from early to late phase is marked by the onset of viral DNA replication (6–18 hpi) and the activity of a virus-encoded RNA polymerase (Grula et al., 1981). Viral DNA replication occurs together with the expression of viral components necessary for the assembly of new nucleocapsids. The newly assembled nucleocapsids are transported from the nucleus to the plasma membrane for budding through GP64-enriched areas, thus originating the so called budded viruses (BVs; Passarelli, 2012). BVs are non-occluded virions surrounded by a plasma membrane-derived envelope containing GP64 as a major structural protein (Washburn et al., 2003). BVs are required for secondary infection: once released, they are transported throughout the hemolymph to infect new cells, a process which, in contrast to what is described for ODVs, is undertaken by GP64 via clathrin-mediated endocytosis (Blissard and Wenz, 1992; Long et al., 2006). In this regard, BVs are the virus form responsible for viral dissemination throughout the host, culminating in a systemic infection. A secondary infection cycle begins with the entrance of BVs into another cell of the insect. After entering the cell, the infection process is similar to what happens in a primary infection, with the nucleocapsids traveling to the nucleus for DNA replication and subsequent viral protein expression. The very late phase of infection (18 hpi) initiates with the expression of proteins that constitute the crystalline matrix of the ODVs, namely polyhedrin. After nucleocapsid assembly, they are enveloped by the polyhedrin matrix to constitute the ODVs. The secondary cycle ends with the extensive infection of larvae tissues and cell lysis, culminating in insect larvae death, and dissemination of ODVs to the environment, where they can remain viable for several years until being ingested by other larvae (Volkman, 1997). Summarizing, BVs and ODVs are genetically identical but differ in their envelope compositions and tissue tropisms, and are produced at different times during infection.

The *in vitro* life cycle of baculovirus is similar to what happens *in vivo*, with a major difference that cultured cells need to be directly infected with the BVs. Since ODVs are forms resistant to environmental factors, there is no need of an occlusion matrix for *in vitro* virus survival. In fact, polyhedrin can be viewed as non-essential for baculovirus *in vitro* cell culture. Given that, recombinant baculoviruses are constructed by replacing the polyhedrin gene (*polh*) by a gene of interest under the control of the very late *polh* promoter (Merrington et al., 1997). Besides the strong activity of *polh* promoter, that allows high productivities of the recombinant protein, it is only expressed in the very late stage of the infection cycle.

## BACULOVIRUS INFECTION: IMPACT ON THE HOST CELL

In the different phases of infection, baculoviruses induce profound changes on host cell properties. For that aim, several virus-encoded proteins interact with host cell factors, altering cellular structures and normal functions, and taking control of cellular gene expression machinery for their own profit (**Table 1**). As a result of such alterations several effects arise: cellular cytoskeleton rearrangement, cell cycle arrest and cytomegaly, apoptosis inhibition, metabolism subversion, and global shut-off of host protein synthesis. Current knowledge on the biology of the proteins involved in the regulation of each of these specific responses are reviewed and detailed below.

**Table 1:** Baculovirus genes affecting host function.

	Baculovirus protein/factors	Host counterparts	Function	Reference
**Cellular adhesion/**	ODV: *pifs* (P74, PIF-1, and PIF-2)	Receptors on midgut epithelial cells	Viral entry through membrane fusion	Faulkner et al. (1997), Kikhno et al. (2002), Fang et al. (2006,2009a), Harrison et al. (2010)
**entry**	BV: GP-64	Receptors on cells	Viral entry by clathrin-mediated endocytosis	Blissard and Wenz (1992), Long et al. (2006)
	VP39, P78, VP80	F-actin	Actin cables formation for nucleocapsids transport across the cytoplasm	Xu et al. (2007), Ohkawa et al. (2010), Marek et al. (2012)
	Arif-1	F-actin	Actin cables accumulation at cell borders	Roncarati and Knebel-Mörsdorf (1997), Dreschers et al. (2001)
	Ie-1, PE38, HE65, Ac004, Ac102, Ac152	G-actin	Drive G-actin accumulation into the nucleus	Ohkawa et al. (2002)
**Cytoskeleton remodeling**	P78/83-C42	G-actin	Arp2/3-mediated nuclear actin polymerization	Braunagel et al. (2001), Wang et al. (2008), Li et al. (2010)
	P10	?	Nuclear integrity	Carpentier et al. (2008)
	EXON0	β-Tubulin	Nucleocapsids engagement to microtubular network and further egress	Fang et al. (2007,2009a)
	Ac93	?	Intranuclear microvesicles formation for virions maturation and egress	Yuan et al. (2011)
	Bm61	?	Egress	Shen and Chen (2012)
	EC27	Cdc2	Cell cycle arrest at G2/M	Belyavskyi et al. (1998)
**Cell cycle arrest**	EC27	Cdk6	Override cellular checkpoints to allow viral DNA replication	Belyavskyi et al. (1998)
	*Lefs*	ATM and/or ATR	Viral DNA replication, shut off of host protein synthesis	Kimberly et al. (2009)
	PK1?; others**	PI3K–Akt/MAPK–ERK–JNK members	Viral DNA replication/late gene expression and progeny production	Reilly and Guarino (1994), Katsuma et al. (2007), Xiao et al. (2009)
**Cellular stress response**	P35**	Effector caspases	Blockage of the apoptotic pathway	Clem et al. (1991), Kamita and Majima (1993), Bump et al. (1995), Bertin et al. (1996), Xu et al. (2001)
	P49**	Initiator and effector caspases	Blockage of the apoptotic pathway	Du et al. (1999), Pei et al. (2002), Zoog et al. (2002), Guy and Friesen (2008)
	IAPs**	?	Inhibition of apoptosis, activation of ubiquitination pathway	Manji et al. (1997), Salvesen and Duckett (2002)
	?**	HSP/HSC70	Viral DNA replication, virions assembly and maturation	Lyupina et al. (2010,^2011^)
**Metabolism**	?	?	Boost in catabolic pathways to fuel infection	Iwanaga et al. (2007), Bernal et al. (2009,^2010^)

### VIRUS ENTRY, INTRACELLULAR TRANSPORT, AND EGRESS OF VIRIONS

Viruses exploit cellular structures in order to be actively transported in the cells. Cytoskeleton proteins have been identified as important factors for viral replication and/or transcription (Fowler, 1990; De et al., 1991). In fact, virus entry, transport, and intracellular localization have been correlated with the reorganization of cytoskeleton proteins (Strauss, 1996; Cudmore et al., 1997). The extent of cytoskeleton reorganization depends on the type of virus, suggesting that a myriad of strategies have co-evolved as a result of the specific interactions established between the virus and its host. Herpesvirus exploits actin and actin-associated myosin motors for viral entry, intranuclear transport of nucleocapsids, and virion egress (Roberts and Baines, 2011). Measles virus induces actin remodeling and microtubule formation upon cell entry, facilitating virus transport into perinuclear spaces, where viral replication occurs, and budding of the newly formed virions (Avota et al., 2011). HIV-1 remodels host cell cytoskeleton in a complex biphasic mode, promoting both inhibition of actin polymerization with looseness of cytoskeleton rigidity in order to favor virus entry, followed by actin remodeling and microtubule network rearrangement for viral cores delivery into the cytoplasm (Stolp and Fackler, 2011).

**FIGURE 1:**
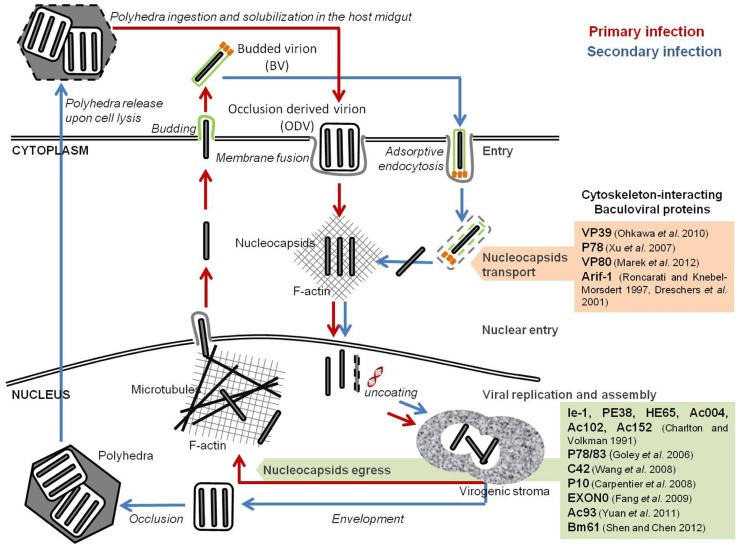
**Baculoviral proteins involved in the remodeling of cellular host cytoskeleton**. Baculovirus infection induces a reconfiguration of the host cytoskeleton for intracellular traveling of nucleocapsids and virions. Nucleocapsids movement into the nucleus occurs through the F-actin cytoplasmic network. The egress of newly formed virions is dependent on a viral-induced actin polymerization inside the nucleus.

The impact of baculovirus infection on host cell cytoskeleton has been studied in detail. Baculoviruses encode several proteins that act in an organized and orchestrated way to remodel the cellular actin network throughout their life cycle (**Figure 1**). Such cytoskeleton rearrangements are of crucial importance for baculovirus infection and proper assembly of newly synthesized virions. In fact, filamentous actin (F-actin) has been shown to be required for viral progeny production of lepidopteran nucleopolyhedroviruses such as *Ac*MNPV, *Spodoptera frugiperda* MNPV (*Sf*MNPV), *Bombyx mori* NPV (*Bm*NPV), *Orgyia pseudotsugata* MNPV (*Op*MNPV), *Lymantria dispar* MNPV (*Ld*MNPV), *Anticarsia gemmatalis* MNPV (*Ag*MNPV), and *Helicoverpa zea* SNPV (*Hz*SNPV; Kasman and Volkman, 2000).

During *Ac*MNPV infection, three major actin cytoskeleton rearrangements occur. First, upon cell entry, F-actin cables can be detected throughout the cytoplasm, often associated with viral nucleocapsids. The formation of these actin cables is independent of viral gene expression, and correlates with the release of nucleocapsids from the endosomes upon internalization of BVs. Fluorescence microscopy studies showed the association of the nucleocapsids with one end of these actin cables, which might indicate the putative role of such structures in the transport of the nucleocapsids to the nucleus (Charlton and Volkman, 1993). Lu et al. (2004) observed that *Helicoverpa armigera* NPV (*Ha*NPV) VP39 nucleocapsid protein not only binds to actin, but also promotes its complexation to form actin cables structures, *in vitro*. In fact, it has been demonstrated that several *Ac*MNPV nucleocapsid proteins (such as VP39, P78, and VP80) bind to actin directly (Xu et al., 2007; Ohkawa et al., 2010; Marek et al., 2012).

After early gene expression, a second alteration of the cytoskeleton occurs, with the formation of F-actin aggregates at the plasma membrane (Charlton and Volkman, 1991; Roncarati and Knebel-Mörsdorf, 1997). Concomitant with the intracellular release of nucleocapsids, actin cables localize at the cell surface and extend into the cytoplasm. At the onset of early gene expression, a rearrangement of the actin network is observed, during which actin cables become more prominent and accumulate at the cell borders (Roncarati and Knebel-Mörsdorf, 1997). This second change is mediated by the product of a single early viral gene, *arif-1* (actin-rearrangement-inducing factor 1), a 47-kDa phosphoprotein that co-localizes with the F-actin aggregates (Dreschers et al., 2001). Expression of *arif-1* alone leads to actin rearrangements comparable to the changes that happen at the late stage of early gene expression (Roncarati and Knebel-Mörsdorf, 1997). Immunofluorescence studies showed that, following *Ac*MNPV infection, Arif-1 co-localizes with F-actin at the plasma membrane until the onset of late gene expression, when Arif-1-induced actin polymerization is no longer detected. In fact, Arif-1 analysis by SDS-PAGE showed that, between 12 and 48 hpi, multiple bands of higher molecular weight appeared, and that phosphatase treatment could reverse this observation. This suggests that Arif-1 is inactivated by phosphorylation (Dreschers et al., 2001).

A third and more profound reconfiguration takes place during late gene expression, when F-actin appears within the nucleus, a feature almost exclusive to baculoviral infection and essential for nucleocapsids morphogenesis (Volkman, 1988; Ohkawa and Volkman, 1999; Kasman and Volkman, 2000). Monomeric globular actin (G-actin) starts to accumulate in the nucleus upon early gene expression. Six early viral gene products are implicated in this process: *ie-1* and *pe38*, both transcriptional activators of several early, late, and very late genes (Blissard and Rohrmann, 1991; Lu and Carstens, 1993; Passarelli and Miller, 1993; Milks et al., 2003); *he65*, a RNA ligase involved in RNA replication, transcription, and modification (Rohrmann, 2011); and *ac004*, *ac102*, *ac152*, which products have not yet been characterized (Ohkawa et al., 2002; Gandhi et al., 2012). Nuclear actin is then polymerized into filaments (F-actin) by the products of late viral genes (Charlton and Volkman, 1991). Time-lapse microscopy studies showed that nuclear recruitment and actin polymerization is a precisely controlled dynamic process. During *Ac*MNPV infection of TN-368 cells, G-actin accumulates in the nucleus between 10 and 20 hpi, and polymerization started 2 h after nuclear entry (Goley et al., 2006a). Such rapid turnover suggests the presence of a regulatory network that commands nuclear actin assembly during baculovirus infection. One such elemental regulator is the cellular Arp2/3 complex, which is activated to nucleate branched actin filaments by proteins called nucleation-promoting factors (NPFs; Welch and Mullins, 2002; Gandhi et al., 2012). In fact, several nucleopolyhedroviruses encode a capsid-associated protein, called p78/83 in *Ac*MNPV, that contains conserved domains of the Wiskott–Aldrich syndrome protein (WASP) family of NPFs (Machesky et al., 2001). However, both the p78/83 protein and the Arp2/3 complex self-localize in the cytoplasm of uninfected cells (Goley et al., 2006a), which points out that another viral protein must participate to recruit both factors to the nucleus. It has been suggested that *Ac*MNPV encoded nucleocapsid protein, C42, a product of a late gene highly conserved among members of the Baculoviridae**family, is responsible for nuclear recruitment of p78/83 and Arp2/3. This protein is present in both BVs and ODVs, possesses a putative nuclear localization signal (NLS) motif and binds to the p78/83 protein in a nucleocapsid-independent manner (Braunagel et al., 2001; Wang et al., 2008; Li et al., 2010). Taken together, the above mentioned cellular and viral factors are believed to act in an orchestrated way to promote actin transport into the nucleus and further polymerization. This step is of paramount importance for proper virion assembly and infectivity, since in the presence of cytochalasin D or latrunculin A, two drugs that interfere with F-actin function, viral progeny production is inhibited, a phenotype observed in several divergent nucleopolyhedroviruses (Kasman and Volkman, 2000).

Also in the late phase of infection, the P10 late viral protein starts to aggregate, forming a thick tubular network surrounding the nucleus that projects into the cytoplasm (Carpentier et al., 2008). During infection the nucleus swells as a result of the accumulation of viral proteins and virions. This P10-associated cage may stabilize the nucleus, preventing its disruption before the ODVs have completely matured and, stabilizing the architecture of cells long enough for the virus to complete its replication process (Carpentier et al., 2008).

Once the virions progeny have properly matured, they need to be transported from the nucleus toward peripheral budding sites. One component that is involved in this egress pathway is EXON0, a conserved structural protein of BV and ODV nucleocapsids found in all lepidopteran alpha-baculoviruses (Fang et al., 2007). Co-immunoprecipitation and confocal immunofluorescence microscopy studies showed that EXON0 interacts with β-tubulin, enabling the “engagement” of BV nucleocapsids with the microtubular network (Fang et al., 2009b). This assists the transport of nucleocapsids in the nucleus from the virogenic stroma to the nuclear envelope and their migration to the plasma membrane. Recently, Yuan et al. (2011) identified a core gene, *ac93*, as an important player in *Ac*MNPV nucleocapsids egress from the nucleus. Mutagenesis assays showed that *ac93* is required for intranuclear microvesicle formation and egress, thus affecting BVs production and ODVs envelopment. Moreover, immunofluorescence microscopy revealed the presence of Ac93 moving toward the cytoplasmic membrane and in the ring zone of the nucleus, late in infection. In fact, this protein was detected in association with the nucleocapsid fraction of both BV and ODV, and the envelope fraction of BV, which further supports its involvement in virion maturation and egress (Yuan et al., 2011). Shen and Chen (2012) identified another core gene, *Bm61*, as a participant in the egress of BVs during *Bm*NPV infection. Deletion of *Bm61* blocked the production of BVs, although DNA replication still occurred. In fact, electron microscopy analysis showed that, despite nucleocapsid assembly still occurred, they remained trapped in the nucleus by impairment of the egress route to the cytoplasm, leading to loss of BVs production. Furthermore, fluorescence microscopy showed the presence of Bm61 at the intranuclear ring zone and nuclear membrane, which illustrates its role in the transport of nucleocapsids to the cytoplasm (Shen and Chen, 2012).

Summarizing, actin and tubulin networks have important roles throughout baculovirus infection. The virus undertakes a successful and dynamic manipulation of cellular cytoskeleton, and these effects span all the infection process from virus entrance and transport to the nucleus, to nucleocapsid formation and egress, which is at the core of successful infection and replication.

### CELL CYCLE ARREST

The cell cycle involves a series of events that take place in a cell leading to its division and duplication. A complete cellular cycle comprises four sequential stages: G1, S, G2, and mitosis. DNA replication and cell division are highly regulated processes that occur at S and M phases, respectively. G1 and G2 are gap phases where the cell prepares itself for the next stage. Progression from one stage to the following is controlled by cyclins and cyclin-dependent kinases (Cdk), which are in turn regulated by a plethora of pathways in response to external stimuli, as well as to the internal conditions of the cell (Morgan, 1995).

A common feature of many viral infections is the subversion of the host cellular cycle to create an intracellular environment suitable for maximized viral DNA replication (Ben-Israel and Kleinberger, 2002; Spink and Fluck, 2003; Chen and Qiu, 2010; Sakai et al., 2011; Li et al., 2012). Very diverse strategies have been developed by viruses, from the stimulation of cell cycle progression in quiescent cells, to blockade of cell cycle progression in proliferating ones (Op De Beeck and Caillet-Fauquet, 1997; Davy and Doorbar, 2007). For instance, HIV Vpr-mediated arrest at G2/M can benefit the early infection stages by increasing the number of integrated proviruses (Groschel and Bushman, 2005). In turn, adenovirus and SV40 polyomavirus infection induces the cell to remain in a pseudo-S phase state, during which normal cellular DNA replication is complete, but the cell still remains competent for viral DNA replication (Lehman et al., 1994, 2000; Ben-Israel and Kleinberger, 2002).

**FIGURE 2:**
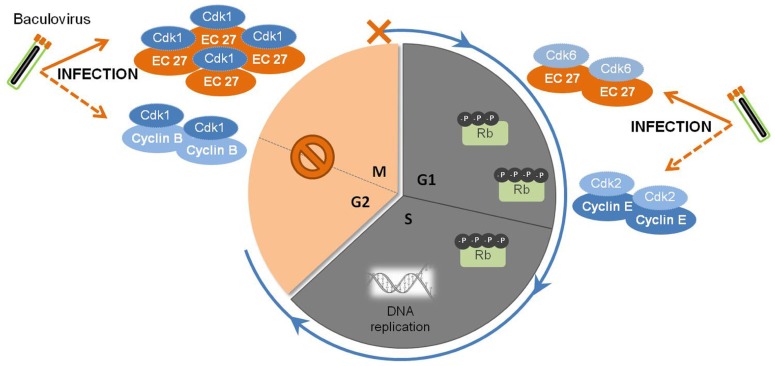
**Baculovirus subversion of host cellular cycle**. Early in infection, the baculovirus causes the augmentation of cellular levels of Cdk1-cyclin B complexes, which are responsible for the regulation of the transition of G2 to M phase. Later on, a viral homolog of host cyclin B, EC27, starts to be expressed and accumulates in the cell. The complex Cdk1-EC27 blocks the cell cycle, and the cells remain arrested In the G2/M phase during the course of infection. Also, EC27 can act in a cyclin D-dependent manner, forming the complex Cdk6-EC27. As a result, the retinoblastoma protein (Rb) becomes phosphorylated, together with the activation of the complex Cdk2-cyclin E. Such phenomena will trigger the components needed for DNA replication, ensuring an environment favorable for viral DNA synthesis and replication.

Baculovirus infection of insect cells causes cell cycle arrest in the G2/M phase (**Figure 2**). Braunagel et al. (1998) showed that when *Sf*9 cells were infected with *Ac*MNPV, approximately 84% of the total cellular population was arrested in G2/M phase by 18–24 hpi. Concomitantly, high levels of Cdc2-associated histone H1 kinase and cyclin B were detected, with the kinase activity remaining detectable through the course of infection. Moreover, this arrest was proven to be necessary for optimal ODVs maturation and assembly since arresting *Sf*9 cells at G1/S boundary by chemical treatment led to abnormal intranuclear microvesicle formation and, consequently, impairment of ODV maturation (Braunagel et al., 1998). During regular cell cycle, cyclin B associates with Cdc2 and this complex is responsible for the progression from G2 to M. The complex accumulates in the nucleus, and once activated by Cdc25, induces a cascade of nuclear architecture rearrangements culminating with the breakdown of nuclear lamina with increased envelope fluidity, therefore committing cells to start dividing. Afterward, cellular cyclin B is degraded and the cell progresses to anaphase (Draetta and Beach, 1989; Nurse, 1994). These findings suggest that early in infection, the cyclin B-Cdc2 complex may be used to regulate the transition from G2 to M phase. However, since only the kinase activity is detectable throughout infection, the prolonged cell cycle arrest during baculovirus infection may be due to a protein(s) encoded by *Ac*MNPV. In fact, *Ac*MNPV was shown to encode a human cyclin homolog protein, the EC27 protein. This protein was described as having a multifunctional operating mode, being able to act in a cyclin B and cyclin D-dependent manner (Belyavskyi et al., 1998). By encoding a cyclin B-like protein, baculoviruses are able to arrest the cellular cycle at G2/M phase maintaining the nuclear architecture at its best for virions progeny assembly and maturation. Actually, the cellular phenotype alterations manifested throughout baculoviral infection, such as enlarged nucleus, increased envelope fluidity, and induction of microvesicles and membranes in the nucleoplasm (Summers and Arnott, 1969; Braunagel et al., 1998), supports EC27 cyclin B-like functions. However, this baculovirus encoded cyclin B does not lead to the breakdown of the nucleus, which remains intact in infected cells, possibly indicating that there are differences with the host protein.

Baculovirus infection of insect cells induces the shut-off of global host protein synthesis by 18 hpi (Carstens et al., 1979; Du and Thiem, 1997), concomitant with the cell cycle arrest at G2/M (Braunagel et al., 1998; Ikeda and Kobayashi, 1999). In fact, beyond this time frame, cellular DNA replication is no longer detected, in opposition to viral DNA replication and gene expression. The presence of an active Cdk6-EC27 complex with cyclin D-like activity can explain this behavior. In non-infected cells, cyclin D association with Cdk6 promotes G1 to S phase transition by phosphorylation of pRb (retinoblastoma protein), a key regulator of the cell cycle (Dowdy et al., 1993; Ewen et al., 1993). Moreover, Cdk-cyclin complexes can “titrate” Cdk inhibitors resulting in activation of the Cdk2-cyclin E complex (Polyak et al., 1994) which then affects components of the pre-initiation complexes to trigger DNA replication (Stillman, 1996). Such observations clearly show that baculovirus encodes specific mechanisms to override cellular checkpoints, which benefits both viral DNA replication and virion assembly and maturation.

### CELLULAR STRESS RESPONSE

Infection is sensed by cells as a stressful situation. Accordingly, viruses trigger diverse cellular responses, including the activation of apoptosis, DNA damage, and heat shock responses (HSR), aimed at fighting the infection by preventing virus replication and dissemination. Consequently, viruses have evolved several strategies in order to circumvent these defense responses. In this section, specific features of baculovirus–host interactions during cellular responses to infection will be discussed. Although these responses are coordinated, for the sake of simplicity, they will be treated separately.

#### Induction of DNA damage response and pro-survival pathways

Several viruses have evolved mechanisms to manipulate the DNA damage response triggered upon viral infection (Chaurushiya and Weitzman, 2010). The cellular response to the presence of damaged DNA is dependent on the activation of two members of the phosphatidylinositol 3-kinase (PI3K) superfamily, the ataxia telangiectasia mutated (ATM) and rad3-related (ATR) proteins (Seviour and Lin, 2010). These systems respond to double-strand breaks in DNA, and single-strand breaks along with stalled replication forks, respectively (Huang et al., 2011). As discussed later on, the downstream effects of the activation of this system include the phosphorylation of numerous effector proteins which function in cell cycle checkpoints, DNA repair, and stimulation of apoptosis. Interestingly, one important downstream substrate of ATM and ATR is P53, which is actively inhibited by many DNA viruses to avoid the inhibition of cell cycle progression and stimulation of apoptosis in response to DNA damage (Huang et al., 2011).

**FIGURE 3:**
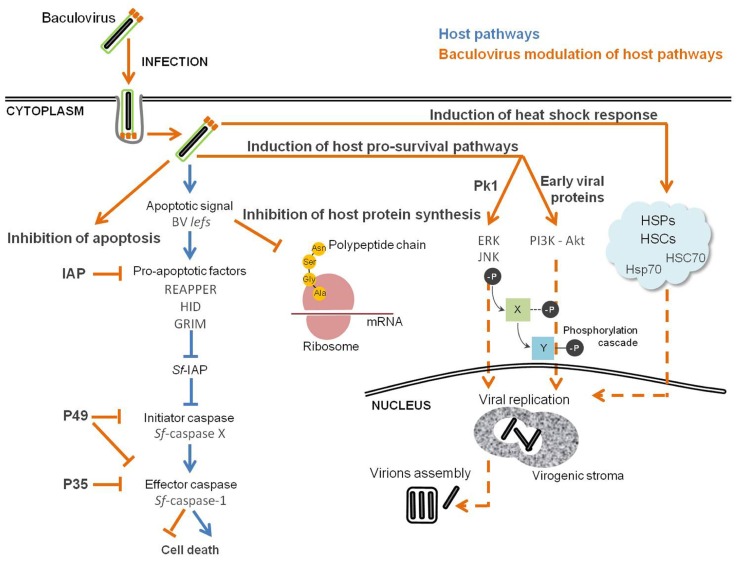
**Baculovirus manipulation of host cellular stress response**. Successful infection is the result of a compromise between host defense response and inhibition of apoptosis, which creates the perfect environment for the replication of the virus. Pathways in “orange” are viral and in “blue” are from the host.

Several mammalian viruses induce a cellular DNA damage response during replication which, in some cases, is required for optimal virus replication. For instance, SV40 efficient DNA replication and virions assembly is dependent on the activation of ATM (Zhao et al., 2008). HSV and HIV-1 also activate ATM, and that ATM signaling is important for viral replication (Lau et al., 2005; Lilley et al., 2005). This is also the case for baculoviruses. In fact, induced apoptosis and shut-off of host protein synthesis are the result of the activation of cellular DNA damage response, and this activation is triggered by viral DNA replication (Clem and Miller, 1994; LaCount and Friesen, 1997; Kimberly et al., 2009). RNA silencing assays directed to genes essential to *Ac*MNPV replication identified the replicative late expression factors (*lef*) as the source of the critical apoptotic signal in infected cells, which also contribute to the inhibition of host cell protein synthesis (Kimberly et al., 2009). Moreover, the addition of ATM and ATR inhibitors to *Sf*9 cultures infected with *Ac*MNPV decreased viral DNA replication and late gene expression (Huang et al., 2011). In this sense, baculovirus control of the cellular response against infection can be viewed as an equilibrium in which the virus takes advantage of the activation of the DNA damage response for DNA replication, shut-off of host protein synthesis to have the machinery available for viral protein expression, together with inhibition of apoptosis by expressing anti-apoptotic factors (**Figure 3**).

Concomitant with the induction of the stress response, viral infection also activates and modulates pro-survival cellular pathways (Cooray, 2004; Ji and Liu, 2008; Furler and Uittenbogaart, 2010; Qin et al., 2011). The mitogen-activated protein kinases (MAPKs), such as extracellular signal-regulated molecule kinase (ERK) and c-Jun NH_2_-terminal kinase (JNK), are involved in the regulation of a plethora of cellular processes, including cell division and control of transcription (Johnson and Lapadat, 2002). The PI3K–Akt pathway controls cell survival and apoptosis, proliferation, migration, and regulates metabolism (Willems et al., 2012). In light of this knowledge, it is not surprising that viruses exploit such cellular pathways to set-up an intracellular environment suitable for efficient replication and progeny production. In fact, hepatitis-B, Epstein–Barr, and vaccinia virus are known to subvert MAPK pathways, a behavior that is behind successful infections (de Magalh aes et al., 2001; Zheng et al., 2003; Gao et al., 2004). During influenza A virus infection, the PI3K–Akt signaling pathway is activated, and inhibition of PI3K results in reduced viral RNA synthesis, protein expression, and viral yield (Shin et al., 2007). Also, human cytomegalovirus (Johnson et al., 2001) and coxsackievirus B3 (Luo et al., 2002; Esfandiarei et al., 2004) depend on the activity of PI3K–Akt and/or MAPK–ERK for efficient replication. It has been previously shown that baculovirus efficient infection is dependent on the activation of both MAPK and PI3K signaling (**Figure 3**). Successful infection of *Bm*NPV is dependent on the activation of ERK and JNK signaling pathways (Katsuma et al., 2007). Western blot assays revealed that ERK and JNK were activated at the late stage of *Bm*NPV infection, and inhibition of both kinases after 12 hpi significantly reduced occlusion body formation and BVs production (Katsuma et al., 2007). Also, late and very late gene expression was impaired, suggesting an important role for ERK and JNK in regulating the *Bm*NPV gene expression program. During *Ac*MNPV infection of *Sf*9 cells the levels of phosphorylated Akt became elevated, and inhibition of PI3K–Akt activation significantly reduced viral yield, BV production, and occlusion body formation (Xiao et al., 2009). The activation of the PI3K–Akt signaling pathway occurs at an early stage of *Ac*MNPV infection, and is of major importance for viral DNA replication. Inhibition of PI3K at 12 hpi not only reduces the level of viral DNA synthesis, but also delays late and very late gene expression. This result is not surprising, since the transition from early to late phase is marked by the onset of viral DNA replication, which in turn occurs together with the expression of very late genes coding for viral structural components necessary for the assembly of new virions (Grula et al., 1981). Taken together, these data suggest that the products of early genes prepare the host cell for viral multiplication, shown here by the activation of PI3K–Akt pathway, ensuring an environment suitable for efficient infection. However, it is not clear which viral proteins are involved in the activation of these signaling pathways. Baculoviral PK1 serine/threonine protein kinase is expressed along with *Ac*MNPV infection and it is essential for *Bm*NPV replication in *Bm*N cells (Reilly and Guarino, 1994; Katsuma et al., 2007). These data suggest that phosphorylation of host and/or viral proteins by PK1 is needed for virus replication, indicating PK1 as a viral candidate for the activation of ERK and JNK kinases. Further studies concerning the identification of the viral counterpart(s) responsible(s) for the activation of these signaling pathways are compulsory.

#### Inhibition of apoptosis

As mentioned before, one of the pathways targeted by the DNA damage response is apoptosis, or programmed cell death, which is a highly regulated process widespread among multicellular organisms. It is characterized by apoptotic body formation, cell shrinkage, membrane blebbing, chromatin condensation and DNA fragmentation, culminating in cellular death (Kerr et al., 1972). Such mechanism is evolutionarily conserved, with the convergence of the apoptotic stimuli to a central pathway that encloses a family of aspartate-specific cysteinyl proteases, i.e., caspases, the main activators and executioners of the apoptotic response. The caspases family is divided in two classes, initiator caspases and effector caspases, which are activated in a sequential manner by auto cleavage in response to apoptotic signaling (Ma and Chang, 2011).

In order to counteract this defense, several viruses have developed mechanisms to block the premature apoptosis of infected cells. These strategies range from mimicking specific cellular regulators, which is the case of the Epstein–Barr virus, African swine fever virus, and herpesvirus, that encode viral homologs for the cellular anti-apoptotic protein Bcl-2, or expressing viral-specific proteins, like the cowpox virus CrmA anti-apoptotic factor (Koyama et al., 2000; Aubert and Blaho, 2001).

Although currently this is observed as a general key strategy, the stimulation of apoptosis by infection was first demonstrated in the baculovirus system (**Figure 3**). Clem et al. (1991) found that baculovirus replication in insect cells triggers apoptosis, and a specific viral gene product, the *Ac*MNPV *p35*, was identified as being responsible for blocking the apoptotic response. In fact, it was later shown that P35 protein is a direct substrate inhibitor of caspases and acts in a stoichiometric manner as a suicide inhibitor (Bump et al., 1995). Caspase-mediated cleavage at the aspartate residue Asp87 within the P35 reactive-site loop leads to the formation of a stable complex covalently bound to the target caspase, subsequently inhibiting apoptosis (Bertin et al., 1996; Xu et al., 2001). In addition to *Ac*MNPV, other baculoviruses have been found to carry *p35* homologs, *Bm*NPV and *Spodoptera litoralis* NPV (*Sl*NPV). The *Bm*NPV *p35* gene has high nucleotide and amino acid sequence identity to the *Ac*MNPV gene, and its presence is needed to inhibit *Bm*NPV induced apoptosis in *B. mori* cells (Kamita and Majima, 1993). The *Sl*NPV *p49* gene encodes a protein with 49% amino acid identity with its *Ac*MNPV homolog *p35* (Du et al., 1999). Computer-assisted modeling and site-directed mutagenesis suggests that the structure of P49 resembles P35, including the presence of a prominent reactive loop that presents Asp94 for cleavage (Pei et al., 2002; Zoog et al., 2002). P49 has the ability to inhibit both initiator and effector caspases, whereas P35 can only inhibit effector caspases. P49 is a substrate inhibitor of the initiator caspase Sf-caspase-X, which is responsible for the proteolysis and activation of Sf-caspase-1 and -2 (Zoog et al., 2002; Guy and Friesen, 2008). This indicates that P49 acts upstream of P35 by inhibiting initiator caspases responsible for the activation of effector caspases.

Another family of baculovirus anti-apoptotic genes is the *iap* (inhibitor of apoptosis) family. These genes are present in genomes from yeasts to humans, and are known to regulate apoptosis and several other cellular functions (Salvesen and Duckett, 2002; Gyrd-Hansen and Meier, 2010). The first *iap* genes described in nature were discovered by complementation assays in *Cydia pomonella* granulovirus (*Cp-iap*) and *Orgyia pseudotsugata* nucleopolyhedrovirus (*Op-iap*; Crook et al., 1993; Birnbaum et al., 1994), in which the *iap *gene compensated for the lack of *p35* in the *annihilator* *Ac*MNPV mutant. At least one *iap* gene is present in almost all the baculovirus genomes sequenced, whereas *p35* genes are present in only a small subset (Clem, 2005). *Op*-IAP was shown to function upstream of P35 in *Sf*21 cells, since its expression was sufficient to block effector Sf-caspase-1 processing, while P35 was not (Manji et al., 1997; Seshagiri and Miller, 1997). However, besides these two examples many of the *iap* genes tested so far do not seem to have the ability to inhibit apoptosis, or this ability is cell type-specific (Bideshi et al., 1999; Maguire et al., 2000). Moreover, given their ubiquitous distribution, maybe their anti-apoptotic activity is restricted to certain scenarios, or they may possess other functions in different viral processes, such as ubiquitin ligases, as shown for other cellular and viral IAPs (Yang et al., 2000; Imai et al., 2003; Green et al., 2004). Structurally, IAP proteins are metalloproteins with one to three copies of a zinc-binding motif, BIR (Baculovirus IAP Repeat), at the N-terminus, and another zinc-binding motif, RING, at the C-terminus (Hinds et al., 1999). BIR-containing motifs of several IAP proteins bind directly a myriad of pro-apoptotic proteins (Miller, 1999). In fact, BIR2 motif of *Op*-IAP interacts with *Drosophila* pro-apoptotic factors REAPPER, HID, and GRIM, and this binding inhibits apoptosis even during overexpression of such factors in *Sf*21 cells (Vucic et al., 1997, 1998a,b). The RING domain is also important, since its removal completely abolishes *Op*-IAP anti-apoptotic activity and strongly diminishes its ability to protect *Sf*21 cells during HID overexpression (Vucic et al., 1998a; Wright and Clem, 2002). RING motif-containing proteins are involved in many cellular functions, ranging from scaffolding of multi-protein complexes (Borden, 2000) to E3 ubiquitin ligase activity (Tyers and Willems, 1999). Actually, the *Op*-IAP RING domain has been shown to have E3 ubiquitin ligase activity, capable of promoting its own and HID ubiquitination, and this ability is of major importance for *Op*-IAP anti-apoptotic properties (Green et al., 2004). Ubiquitination is a post-translational modification that can have different effects on the targeted substrate, such as targeting to proteasome-dependent proteolysis or the modulation of protein function, structure, assembly, and localization (Deshaies and Joazeiro, 2009). The relevance of this activity for the *in vivo* anti-apoptotic function of *Op*-IAP is still unclear. Direct interaction and subsequent modulation of pro-apoptotic factors by IAP proteins is evident, and further studies are encouraged in order to disclose the peculiarities of such dynamic cross talk.

#### Induction of the heat shock response

A hallmark of universal cellular defense to various environmental and pharmacological stresses is the activation of the HSR (Gidalevitz et al., 2011). The induction of HSR leads to the rapid and robust expression of members of the chaperone family of heat shock proteins (HSPs) and respective cognates (HSCs), in order to protect the cell from proteotoxic stresses and to maintain protein homeostasis (Fujimoto and Nakai, 2010).

HSP70s and HSP90s, members of the HSPs family, are involved in the replicative cycles of DNA and RNA viruses. Not only have they been identified in the regulation of viral gene expression *via* interaction with specific viral proteins, but they also participate in capsid assembly and disassembly (Mayer, 2005; Xiao et al., 2010; Nagy et al., 2011). Such observations demonstrate that viruses also exploit HSR as an infection strategy. A proteomic study by 2D-GE coupled with mass spectrometry showed that host HSC70 was associated with ODV of *Bm*NPV, suggesting a possible involvement of HSP during the assembly of baculoviral virions (Liu et al., 2008). Nobiron (2003) used a differential display approach to search for host mRNA transcripts that could be up-regulated during *Ac*MNPV infection of *Sf*9 cell line. They found one transiently up-regulated transcript encoded by *hsc70*, for which expression peaked at 6 hpi. Similarly, a microarray approach to analyze the global transcriptional profile of infected *B. mori* cells showed that the *hsc70* ortholog was up-regulated during infection, and this increase was detected only until 24 hpi (Sagisaka et al., 2010). A combined microarray assay complemented with qRT-PCR was applied for *Sf*21 transcriptome analysis during the infection with *Ac*MNPV (Salem et al., 2011). Despite the fact that the majority of cellular genes were down-regulated during the course of infection, the expression of two members of the *hsp70 *family were augmented. Lyupina et al. (2010) monitored the induction of HSPs of the 70-kDa family (HSP/HSC70) in *Sf*9 cells after infection with *Ac*MNPV by Western blot analysis. The authors reported that *Ac*MNPV infection induces and stimulates several HSP70s, and that the infection process markedly potentiates HSR by boosting the HSP/HSC70s content (**Figure 3**). The use of chemical inhibitors of HSR decreased the rate of viral DNA synthesis, providing experimental evidence of the importance of such pathway for baculovirus replication. Moreover, HSP70s co-localize with ubiquitinylated proteins in speckles in the cytoplasm of *Ac*MNPV-infected cells, forming aggresome-like structures that can contain proteins for digestion and/or sequestration during infection (Lyupina et al., 2011). In fact, the ubiquitin-proteasome system is required during *Bm*NPV infection, since its inhibition resulted in reduced BV and ODV formation, together with the suppression of polyhedrin expression (Katsuma et al., 2011). Ubiquitin homologs are found in most lepidopteran baculovirus genomes (Katsuma et al., 2008). Although non-essential for viral replication, the baculovirus ubiquitin protein (*v*-UBI) is involved in the formation of *Ac*MNPV viral particles, since its loss resulted in a reduction of BV production (Reilly and Guarino, 1996). Additionally, biochemical experiments suggest a putative role for *v*-UBI in the blocking of the host degradative pathways of short-lived protein(s) during infection (Haas et al., 1996). Taken together, these data suggest a close collaboration of HSPs and ubiquitin-proteasome system during the baculovirus replicative cycle. Studies exploring the interactome profile between viral proteins and factors triggered upon stress induction are strongly encouraged, in order to identify the players and mechanisms responsible for balancing host damage and stress response.

### METABOLISM

Viral infection claims an intensification of host cell biosynthetic activity in order to supply building blocks needed for the biogenesis of membrane lipids and for the synthesis of viral nucleic acids and proteins (Munger et al., 2006, 2010). In fact, viruses are considered as “metabolic engineers” (Maynard et al., 2010). In this regard, the success of infection is highly dependent on the metabolic state of the cells at the moment of infection, jointly with the viral manipulation of energy metabolism to fit such needs (Carinhas et al., 2010, 2011a). Despite many recent reports, the characterization of insect cell metabolic response to baculovirus infection is still at its infancy.

**FIGURE 4:**
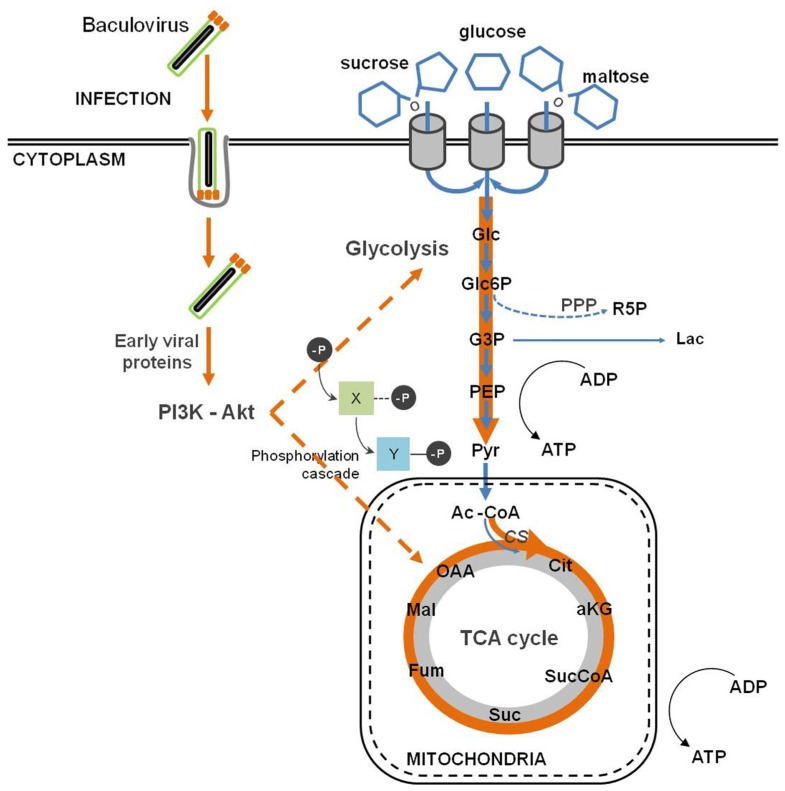
**Baculovirus infection impact on host cell metabolism**. Baculovirus infection causes an intensification of glycolytic and tricarboxylic acid cycle (TCA) fluxes, which indirectly follows the activation of the PI3K–Akt signaling pathway.

Baculovirus infection provokes an important metabolic burden on insect cells, causing an enhancement of the fluxes through the major catabolic pathways, namely glycolysis and tricarboxylic acid cycle (TCA; Bernal et al., 2009) as reflected in the changes in fluxes and enzyme activities after infection (Bernal et al., 2010; **Figure 4**). An increase in the oxygen uptake rate is also observed, which accounts for a higher rate of respiration upon infection (Kamen et al., 1996; Palomares et al., 2004; Bernal et al., 2009). Moreover, there is a drop in systems productivity when insect cells are infected with baculovirus at high cell densities, the so called “cell density effect.” Bernal et al. (2009) succeeded in deciphering the metabolic basis of such phenomenon, during which *Sf*9 cells undergo a progressive inhibition of central metabolism (Bernal et al., 2009). Since a successful viral infection strongly correlates with the energetic state of the cell, viral replication is impaired in high cell density cultures. Iwanaga et al. (2007) performed an exploratory analysis using subtractive hybridization in order to identify differentially expressed host genes following *Bm*NPV infection. The authors paid special attention to the response of energy metabolism to infection, and reported the up-regulation of *citrate synthase* and *ATP-dependent proteasome 26S *homologous genes. Citrate synthase is the first enzyme of the TCA cycle, which plays a central role in aerobic energy production and metabolic interconversions in mitochondria (Holloszy et al., 1970). On the other hand, the proteasome-ubiquitin pathway plays an important role during baculovirus infection as mentioned previously (Katsuma et al., 2011). Next-generation sequencing and gene enrichment analysis also showed that gene sets related to mitochondrial function were highly up-regulated during the course of *Bm*NPV infection of *Bm*5 cells (Xue et al., 2012). A recent high-throughput analysis of baculovirus proteomic responses of *Sf*9 cells to infection with *Ac*MNPV identified two up-regulated metabolic enzymes, pyruvate dehydrogenase and aldehyde dehydrogenase (Carinhas et al., 2011b). Therefore, the up-regulation of such cellular proteins indicates that baculoviruses manipulate host energy metabolism to fuel its own replication and further envisages the importance of energy metabolism in supporting infection.

All these alterations described in central metabolism are linked to the host–virus interactions described in the previous sections. Baculovirus replication depends on the activation of PI3K–Akt signaling pathway (Xiao et al., 2009). One of the downstream targets of Akt is the adenosine 5′ monophosphate (AMP)-activated kinase (AMPK), a key sensor which regulates the metabolic status of cells (Cantó et al., 2009). In fact, it is activated by an increase in the AMP:ATP ratio (i.e., a low energy charge) and balances energy homeostasis by up-regulating catabolic processes while inhibits anabolism. A similar regulatory mechanism is activated in other viruses (Terry et al., 2012). In addition, both *Ac*MNPV and *Bm*NPV encode nucleotide diphosphate hydrolases belonging to the Nudix family. The corresponding genes are expressed early in infection and are essential for the replication of the virus (Ge et al., 2007; Chen et al., 2010). The essentiality of this activity is intriguing, although several metabolic regulatory roles have been proposed for nucleotides and related metabolites, which might contribute to the observed effects of infection (Tong and Denu, 2010).

## PROTEOMIC ANALYSIS OF BACULOVIRUS–HOST CELL INTERACTIONS

As seen along the previous sections, viral infection induces profound alterations on the physiology of the host cells, and the latter respond to such changes by translating a complex network of protein–protein interactions and biochemical signaling events into functional responses. Systems level knowledge about the host effectors involved in the response to virus infection and the functions induced by viruses is crucial to understand the molecular basis of viral pathogenesis at a whole cell/organism level.

In addition to their well proven biotechnological and biomedical potentials, the study of the molecular biology of baculovirus infection has also offered meaningful insights into conserved viral mechanisms of manipulation and subversion of the host cell (Clem, 2001; Goley et al., 2006b).

Despite the advances in the so-called *omic* technologies, their application to the baculovirus–host system has been held back by the lack of sequenced host genomes, and the scarcity of curated databases. While complete genome sequences for more than 53 baculovirus are available (van Oers, 2011), currently, the only fully sequenced insect host is *B. mori *(Xia et al., 2004). Global gene expression profiles of *S. frugiperda* and *B. mori* derived cell lines have been recorded at various time-points, following baculovirus infection (Nobiron, 2003; Iwanaga et al., 2007; Sagisaka et al., 2010; Salem et al., 2011). Also, the temporal gene expression programs of the viral genes of several members of the Baculoviridae family (*Ac*MNPV, *Tn*SNPV, *Bm*NPV) have been elucidated during their respective infection cycles (Yamagishi et al., 2003; Iwanaga et al., 2004, 2007; van Munster et al., 2006). Even scarcer are large scale proteomic studies. Popham et al. (2010) performed a proteomic analysis based on 2D-GE-MS/MS to examine the protein expression of permissive and non-permissive insect host cells (*H. zea* and *Heliothis virescens* derived cell lines, respectively) for *Ac*MNPV. The authors identified 18 differentially expressed proteins after 24 hpi in the permissive cell line, among which there were members of signal transduction pathways, protein function and homeostasis, and cell survival (Popham et al., 2010). Recently, Carinhas et al. (2011b) applied a SILAC approach for quantitative proteomics of *Sf*9 cells during growth and early baculovirus infection, contributing with the first comparative quantitative proteomic analysis of the response of *S. frugiperda* cells to infection. The lack of an annotated genome sequence was overcome by cross-referencing to a database that included sequences of proteins from *S. frugiperda* and related insect species. The authors found new differentially expressed proteins related to energy metabolism, endoplasmic reticulum and oxidative stress during *Ac*MNPV infection (Carinhas et al., 2011b). In particular, the up-regulation of two metabolic enzymes, PDH-E3 and ALDH, was observed, which account for an increased efficiency of the coupling of glycolysis and the TCA cycle and for the anaplerotic feeding of carboxylic acids, respectively. These observations go along with the increased metabolic fluxes of central carbon metabolism during baculovirus infection (Bernal et al., 2009), emphasizing the importance of energy metabolism during viral infection. Concerning the cellular stress response to infection, the authors observed a decrease in the levels of the chaperone ERp57 and the polypeptide transporter SRP57. Both proteins are effectors of the untranslated-protein response (UPR), and their down-regulation envisages the baculovirus capacity in avoiding the deleterious effects of cellular stress response.

Altogether, these results demonstrate that efficient replication of baculovirus depends on the capacity of viral manipulation of different cellular pathways, the control of which are needed for the success of viral infections.

Systems-level knowledge of baculovirus–host interactions requires an effort from the scientific community in the direction of insect cells/host genome sequencing. Recent efforts by Nguyen et al. (2012) succeeded in the transcriptome sequencing of *H. zea* cell line, providing a microarray platform to investigate baculovirus–insect cell interactions. Also, Xue et al. (2012) used next-generation sequencing to analyze differential gene expression following *Bm*NPV infection of *Bm*5 cell line. A gene enrichment analysis showed that gene sets enclosing cytoskeleton, transcription, translation, energy metabolism, iron metabolism, and ubiquitin-proteasome pathways are altered during the course of baculovirus infection. The information gathered during transcriptional analysis was then used to define the interactome network between *Bm*NPV and host proteins at a systems level, revealing direct interaction of viral proteins with cellular components, such as the proteasome, the cytoskeleton and the spliceosome (Xue et al., 2012).

## CONCLUSION

Baculoviruses have developed several strategies to subvert the mechanisms of cellular defense against infection. In this fight, baculoviruses take control of cellular structures, such as the cytoskeleton, and trigger signaling responses leading to cell cycle arrest, induction of the DNA damage response, inhibition of apoptosis and intensification of energy metabolism. Although many of these responses are shared by different viruses, the specific characteristic features of the adaptations evolved by Baculoviruses have been highlighted in this review.

Although many studies concerning baculovirus molecular biology and the infection process have been pursued, there is a lack of a whole-system level integration of cross-platform reported data. Thus far, the application of high-throughput transcriptomic and proteomic approaches has been hampered by the current unavailability of lepidopteran genome sequences.

System-level proteomic studies can provide useful insights on the dynamic host response to infection. Indeed, the direct cross-talk between virus and host occurs mainly at the protein level. Viral proteins are major effectors on the subversion and manipulation of host cell physiology. Mapping such intricate network of interactions and, more importantly, deciphering their outcome at the cellular level, will provide a step-further in our current knowledge on the biological outcome of infection.

## Conflict of Interest Statement

The authors declare that the research was conducted in the absence of any commercial or financial relationships that could be construed as a potential conflict of interest.
